# Influenza vaccination is associated with a decreased risk of atrial fibrillation: A systematic review and meta-analysis

**DOI:** 10.3389/fcvm.2022.970533

**Published:** 2022-10-20

**Authors:** Menglu Liu, Weichun Lin, Tiangang Song, Huilei Zhao, Jianyong Ma, Yujie Zhao, Peng Yu, Zhiwei Yan

**Affiliations:** ^1^Department of Cardiology, Seventh People’s Hospital of Zhengzhou, Zhengzhou, China; ^2^Department of Gastroenterology, The Third Affiliated Hospital, Sun Yat-sen University, Guangzhou, China; ^3^Department of Endocrinology and Metabolism, The Second Affiliated Hospital of Nanchang University, Nanchang, China; ^4^Department of Anesthesiology, The Third Hospital of Nanchang, Nanchang, China; ^5^Department of Pharmacology and Systems Physiology, College of Medicine, University of Cincinnati, Cincinnati, OH, United States; ^6^Department of Sports Rehabilitation, College of Human Kinesiology, Shenyang Sport University, Shenyang, China

**Keywords:** influenza vaccination, arrhythmia, atrial fibrillation, ventricular arrhythmia, meta-analysis

## Abstract

**Background:**

Evidence from longitudinal studies has shown that influenza infection is linked to an increased risk of arrhythmia. Therefore, we aimed to assess the role of influenza vaccination in arrhythmia prevention.

**Materials and methods:**

The PubMed, Embase, and Cochrane Library databases were searched to identify studies that investigated the potential effects of the influenza vaccine on arrhythmia risk published until October 25th, 2021. The study was registered with PROSPERO (CRD42022300815).

**Results:**

One RCT with 2,532 patients and six observational studies with 3,167,445 patients were included. One RCT demonstrated a non-significant benefit of the influenza vaccine against arrhythmias [odds ratio (OR) = 0.43, 95% confidence interval (CI): 0.11–1.64; *P* = 0.20] in patients after myocardial infarction or those with high-risk stable coronary heart disease. A meta-analysis based on observational studies showed that vaccination was associated with a significantly lower risk of arrhythmia (OR: 0.82, 95% CI: 0.70–0.97; *P* = 0.02; *I*^2^ = 76%). Additionally, subgroup analysis showed a decreased risk of atrial fibrillation (AF) (OR: 0.94, 95% CI: 0.90–0.98; *P* = 0.006; *I*^2^ = 0%) and a non-significant but positive trend concerning ventricular arrhythmias (VAs) (OR: 0.68, 95% CI: 0.42–1.11; *P* = 0.12; *I*^2^ = 85%) after influenza vaccination.

**Conclusion:**

Based on the current evidence, influenza vaccination may be associated with a reduced risk of arrhythmia, especially AF. Influenza vaccination may be an effective tool for the prevention of arrhythmias. The effect of influenza vaccination on the risk of VAs and arrhythmias in patients at low risk for cardiovascular diseases should be further studied.

**Systematic review registration:**

[https://www.crd.york.ac.uk/PROSPERO/], identifier [CRD42022300815].

## Introduction

Cardiac arrhythmias, including atrial fibrillation (AF), atrial flutter, ventricular flutter, ventricular fibrillation, and heart arrest, are a common kind of cardiovascular disease, resulting in frequent hospitalizations, hemodynamic abnormalities, and thromboembolic events. Various infections, either by bacteria or viruses, can cause cardiac injury and secondary dysfunction of the cardiac conduction system (1–5). Among these viruses, influenza virus infection has a high incidence. It has been estimated that ∼3–11% of individuals in the United States have symptomatic influenza each year, with an average incidence of 5.1% in adults and 8.7% in children (6). An increased occurrence of influenza-associated cardiac arrhythmias, including atrial arrhythmia, cardiac conduction system abnormalities, ventricular arrhythmia (VA), and atrioventricular block, has been reported in previous studies (7–10). Consequently, the flu vaccine could be an effective tool in preventing arrhythmias. However, in 2002, the Canadian Adverse Events Following Immunization Surveillance System (CAEFISS) listed AF as one of many adverse events reported following vaccination against influenza (11). Since then, whether the influenza vaccine reduces the risk of arrhythmias has remained unclear: some studies have found a negative association (12, 13), whereas other studies have found a positive association (14). Although the relationship between the influenza vaccine and cardiovascular events is still being investigated, the 2019 European guidelines for the secondary prevention of cardiovascular diseases have included influenza vaccination as a class I, level of evidence B recommendation to prevent cardiovascular diseases in patients with coronary and other atherosclerotic vascular diseases (15). The European guidelines do not explicitly link the flu vaccine to cardiac arrhythmias.

Given this background, it is not clear whether influenza vaccination is associated with reduced cardiac arrhythmias. Thus, in the present study, we aimed to (1) clarify the relationship between the flu vaccine and cardiac arrhythmias and (2) further explore the associations of specific types of arrhythmias (e.g., AF and VA) with the flu vaccine.

## Methods

This study was performed according to preferred reporting items for systematic reviews and meta-analyses (PRISMA) guidelines (Supplementary Table 1).^1^ The protocol was registered with PROSPERO (International Prospective Register of Systematic Reviews. https://www.crd.york.ac.uk/PROSPERO/ -registration number-CRD42022300815).

### Literature search

Two authors (ML and WL) independently searched the PubMed, Embase and Cochrane Library databases for published articles without language restrictions through October 25th, 2021. The following MeSH terms were used for all databases: (“Influenza vaccination”) AND (“Arrhythmias” OR “Atrial Fibrillation” OR “Atrial Flutter” OR “Ventricular Fibrillation” OR “Ventricular Flutter” OR “Ventricular Tachycardia” OR “Heart Arrest”). We also checked the conference abstracts and bibliographies of related literature to obtain other articles that might meet the requirements. Any discrepancy was resolved through discussion (ML and WL) until a consensus was reached.

### Study selection

Studies were included if they met the following criteria: (a) were designed as randomized controlled trials (RCTs) or observational studies; (b) assessed the relationship between influenza vaccination and the risk of arrhythmia; and (c) reported estimate effects as adjusted relative risks (RRs)/hazard ratios (HRs)/odds ratios (ORs) with the corresponding 95% confidence intervals (CIs) or other measures that could be used to compute these values.

According to the population, intervention, comparison, outcome, and study design (PICOS) framework, the inclusion criteria were as follows: (1) Participants: adults (aged > 18 years); (2) Exposure and comparator: vaccinated vs. Unvaccinated people or those beyond the period of efficacy after influenza vaccination; (3) Outcomes: the risk of arrhythmia (we did not restrict the type of arrhythmia, including AF, atrial flutter, ventricular fibrillation, ventricular flutter, cardiac arrest) and estimate effects reported as RRs/HRs/ORs with the corresponding 95% CIs or other measures that could be used to compute these values; and (4) Types of studies: RCTs or observational studies. We selected the most recent publication if multiple studies used the same population. Certain publication types (animal studies, conference abstracts, editorials, letters, and reviews) or studies with unavailable data were excluded from this analysis.

### Data extraction and quality assessment

The study information and the basic characteristics of the articles were extracted, including the first author, year of publication, country, study design, study participants, sample size, age, sex%, method of vaccine exposure, length of follow-up, reported outcomes, risk estimates, 95% CIs and adjustments. The Newcastle-Ottawa Scale (NOS) was used to evaluate the quality of the included observational studies. Studies with an NOS score > 6 were considered high-quality studies.

Review Manager (RevMan) (version 5.4, Cochrane Collaboration) software and Stata software (Version 16.0, StataCorp LP, College Station, TX, USA) were applied for analysis. ORs and 95% CIs were used to estimate the effects. The *I*^2^-test (*I*^2^ < 50%, *I*^2^ = 50–75%, and *I*^2^ > 75% represent low, moderate, and high heterogeneity, respectively) was used to represent the degree of heterogeneity across the studies (16). The presence of heterogeneity was measured by the *Q*-test, with *P* < 0.10 considered statistically significant. Subgroup analyses were stratified by types of arrhythmias and study design.

The random-effects model was applied considering the potential heterogeneity. Egger’s test and Begg’s test were used to detect publication bias. A two-tailed *P* < 0.05 was considered statistically significant.

## Results

### Study selection

**FIGURE 1:**
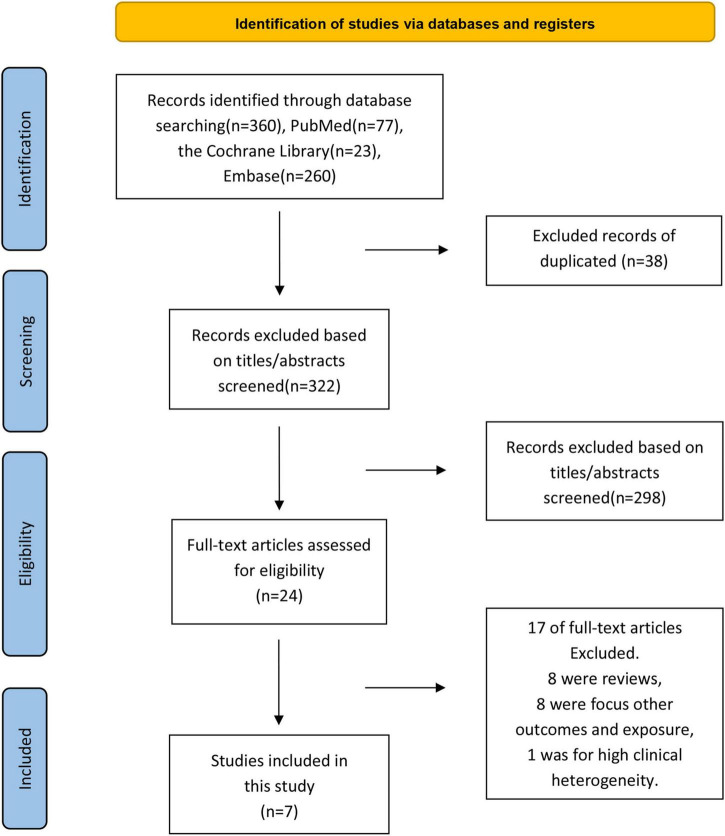
Flowchart of included and excluded studies in the meta-analysis of the association between influenza vaccination and the risk of arrhythmia.

A systematic search of online databases revealed 360 articles (PubMed = 77, EMBASE = 260, Cochrane Library = 23). After excluding duplicates and screening the titles/abstracts, 24 articles were subjected to a more detailed full-text assessment, after which seven articles with 3,169,977 patients were included in the analysis (12–14, 17–20) (Figure 1). The excluded studies and the reasons for their exclusion are listed in Supplementary Table 2.

### Study characteristics and quality

The basic characteristics of the included studies are shown in Table 1. Overall, among the seven studies included, one was an RCT (20), and six were observational studies [cohort studies (12, 14, 18, 19) = 4, case–control studies (13, 17) = 2]. The publication years ranged from 2000 to 2021, the mean age of participants ranged from 59 to 73.3 years, and the sample sizes ranged from 229 to 2,957,091. Two studies focused on a composite of arrhythmic events (18, 20), two studies focused on the risk of AF (14, 17), and the remaining three studies focused on the risk of VA (12), the use of implantable cardioverter defibrillator (ICD) therapies (19), and primary cardiac arrest (13). Most of the observational studies had NOS scores > 6, except one (12), indicating that most studies were of acceptable quality (Supplementary Table 3). All studies reported adjusted risk estimates. Age was the only variable for which all the observational studies adjusted their findings, except the study of Chen et al. (12). The determination of influenza vaccination was defined as receiving an influenza vaccination during the previous 12 months via the medical database in two studies (13, 17), receiving at least 1 dose during the follow-up period (18) or study period (14) via the medical database in two studies, and the self-reported use of influenza vaccination in the previous influenza season in the study of Singh et al. (19). One study did not mention the definition of influenza vaccination (12). The RCT involved nurses providing vaccinations to participants during the study period to determine whether they had received an influenza vaccination (20). Additionally, all studies identified arrhythmia using the International Classification of Diseases, Clinical Modification codes, except the study of Chen et al. (12), which did not clarify how arrhythmia events were identified.

**TABLE 1:** Characteristics of included studies in this meta-analysis.

First author, year, country	Study design	Study participants	Sample size	Age (years), male	Determine method of vaccine exposure	Mean or median duration of follow-up (year)	Outcomes reported	Effect size/95% CI	Adjustments
**Observational studies**									
Chang et al. (17) Taiwan	Case-control study	–	56,870	70.9, 55.72%	Vaccinated 1 year before the enrollment	NA	Risk of AF	OR 0.98 (0.77, 1.26)*	Age, gender, medical history (hypertension, DM, congestive HF, MI, PAD, COPD, ESRD, ischemic stroke/TIA, hemorrhagic stroke, GERD, sleep apnea, cancer, dyslipidemia, dementia, major depression, autoimmune diseases, liver cirrhosis, statin use, medical utilization)
Chen et al. (12) China	Retrospective cohort study	Patients with COPD	18,658	NA	NA	1	Risk of VA	HR 0.53 (0.39, 0.72)	Year of diagnosis
Modin et al. (18) Denmark	Retrospective cohort study	Diagnosed with HF	134,048	73.3, 55.9%	Vaccinated at least 1 dose within the follow-up period	3.7	Risk of arrhythmia Risk of AF or flutter Risk of VA	HR 0.95 (0.89, 1.01)* HR 0.94 (0.90, 0.99) HR 1.03 (0.88, 1.21)	Age, gender, socioeconomic status, education level, comorbidities, drugs with prognostic impact in HF, inclusion year.
Singh et al. (19) Canada	Retrospective cohort study	Patients with past or probable arrhythmias	229	69.72, 85.29%	Self-report influenza vaccination in the previous influenza season	9 months	Occurrence of arrhythmia requiring ICD therapies	RR 0.83 (0.18, 3.79)	Age, comorbidities, history of ventricular arrhythmias.
Siscovick et al. (13) Washington	Case-control study	–	549	59 years, 80%	Self-report influenza vaccination during the previous 1 year	NA	Risk of primary cardiac arrest	OR 0.51 (0.33, 0.79)	Age, gender, current smoking, former smoking, hypertension, diabetes, weight, height, habitual physical activity, saturated fat intake, family history of myocardial infarction or sudden death, educational attainment, employment, and general health status.
McNeil et al. (14) America	Retrospective cohort study	USA military	2,957,091	≥18 years old, 84.66%	Vaccinated at least 1 dose during study period	3.6	Risk of AF	RR 0.94 (0.84, 1.04)	Gender, race, ethnicity, age, and military service characteristics.
**RCTs**									
Fröbert et al. (20) Europe	RCT	Patients shortly after myocardial infarction or with high-risk stable CAD	2,532	59.85, 80.51%	Vaccinated during the trial and not received influenza vaccination during the ongoing influenza season	1	Hospitalization for arrhythmia	HR 0.43 (0.11, 1.64)	Age, gender, MI, stable coronary artery disease, BMI, diabetes, smoking status, hyperlipidemia, hypertension, previous MI, previous PCI, previous CABG, number of diseased vessels.

*Obtained by merging extracted data. CI, confidence interval; NA, non-available; AF, atrial fibrillation; OR, odd ratio; HR, hazard ratio; RR, relative risk; DM, diabetes mellitus; HF, heart failure; MI, myocardial Infarction; PAD, peripheral arterial disease; COPD, chronic obstructive pulmonary disease; ESRD, end-stage renal disease; TIA, transient ischemic attacks; GERD, gastroesophageal reflux disease; VA, ventricular arrhythmia; ICD, implantable cardioverter defibrillator; USA, United States; RCT, randomized controlled trial; BMI, body mass index; PCI, percutaneous coronary intervention; CAD, coronary artery disease; CABG, coronary artery bypass grafting.

### Association between influenza vaccination and the risk of arrhythmia

**FIGURE 2:**
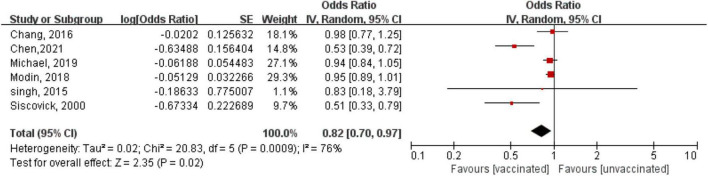
Forest plot for the association between influenza vaccination and the risk of arrhythmia. The diamond indicates the pooled estimate. Red boxes are relative to study size, and the black vertical lines indicate the 95% CIs around the effect size estimate.

One RCT (20) with 2,532 patients and six observational studies (12–14, 17–19) with 3,167,445 patients assessed the relationship between influenza vaccination and the risk of arrhythmia. The RCT (20) showed a trend of a reduced risk but not a significant benefit of hospitalization for arrhythmia (OR = 0.43, 95% CI: 0.11–1.64; *P* = 0.20) in patients after myocardial infarction or those with high-risk stable coronary heart disease after 12 months of follow-up. A meta-analysis based on observational studies showed that compared with the unvaccinated group, in the vaccinated group, the risk of arrhythmia decreased by 18% (OR = 0.82, 95% CI: 0.70–0.97; *P* = 0.02), with high heterogeneity (*P* = 0.0009, *I*^2^ = 76%) (Figure 2).

**FIGURE 3:**
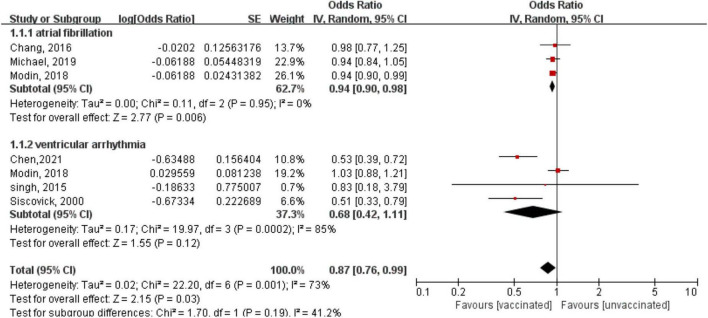
Forest plot for the association between influenza vaccination and the risk of arrhythmia, stratified by the type of arrhythmia. Atrial fibrillation, ventricular arrhythmia.

A subgroup analysis showed a reduced risk of AF with influenza vaccination (OR = 0.94, 95% CI: 0.90–0.98; *P* = 0.006), with low heterogeneity (*P* = 0.95, *I*^2^ = 0%) (Figure 3); however, there was a non-significant risk of VA (OR = 0.68, 95% CI: 0.42–1.11; *P* = 0.12), with high heterogeneity (*P* = 0.0002, *I*^2^ = 85%) (Figure 3). We also conducted a subgroup analysis stratified by study design. The results showed that there was no subgroup difference in the study design (*P* for subgroup difference = 0.65), although there was a borderline reduced risk of arrhythmia in the retrospective cohort studies (OR: 0.85, 95% CI: 0.71–1.01; *P* = 0.06; *I*^2^ = 78%), but there was no statistically significant difference in the case-control studies (OR: 0.73, 95% CI: 0.0.38–1.37; *P* = 0.32; *I*^2^ = 85%) (Supplementary Figure 2).

### Publication bias

As shown in Supplementary Figure 1, Begg’s test (*P* = 0.452) and Egger’s test (*P* = 0.222) showed no statistically significant potential publication bias, although correcting for publication bias was not recommended considering that the number of included studies was limited (*N* < 10).

## Discussion

### Main findings

Although an RCT reported a non-significant association between influenza vaccination and arrhythmias, pooled results from observational studies showed that the risk of arrhythmia decreased by 17% in vaccinated individuals compared with unvaccinated individuals, thereby demonstrating that influenza vaccination might play an important role in the prevention of arrhythmia. Subgroup analysis showed that influenza vaccination decreased the risk of AF by 6%. To our knowledge, this is the first meta-analysis to examine the association of influenza vaccination with the risk of arrhythmia.

Pooled results from observational studies showed a decreased risk of arrhythmia with influenza vaccination; however, an RCT showed a non-significant benefit. The inconsistent results between the RCT and observational studies should be interpreted with caution. Several reasons may account for this inconclusive result. First, arrhythmias were a secondary endpoint in the RCT, and thus, the effect of influenza vaccination on arrhythmias might be below the power to detect a significant difference. Second, there was a very large magnitude of effect (OR = 0.43, 95% CI: 0.11–1.64) for arrhythmias after influenza vaccination in the RCT. We cannot exclude the possibility that the risk estimates would reach significance if more patients or studies were included. Finally, the duration of the trial was only 12 months, which was relatively short and might have an impact on the outcome of hospitalization rates for arrhythmias. Overall, we supposed that the influenza vaccine may benefit against arrhythmias.

The subgroup analysis showed that there was a significant benefit for AF rather than VA. The incidence of VA is much lower than that of AF, which might reduce the statistical power to detect a significant difference in the benefit against VA. Moreover, the measure of VA varied among the studies; two studies defined VA as the use of ICD therapies, one study defined VA as primary cardiac arrest, and one study did not state the definition clearly. The above point might be responsible for the non-significant association. Interestingly, the study of Chen et al. (12). Demonstrated dose-dependent protective effects of influenza vaccination against VA. After adjustment, they showed that individuals vaccinated more than four times during the follow-up period showed a low risk of developing VA during the influenza season compared to those vaccinated one time [adjusted hazard ratio (aHR): 0.61] and 2–3 times (aHR: 0.65) (12). Therefore, further prospective studies with larger sample sizes are needed to assess the influenza vaccination-associated risk of VA.

### Comparison with previous studies

Our results appear to agree with those from previous studies. Evidence from several clinical trials has shown the benefit of influenza vaccination on the incidence rates of cardiovascular events or mortality in patients with cardiovascular disease (21–23). The FLUVACS study, published in 2004, randomly assigned 200 patients with myocardial infarction admitted within the previous 72 h and 101 patients scheduled for angioplasty/stenting (PCI) to a vaccinated or unvaccinated group and found that influenza vaccination reduced the risk of death and ischemic events in patients with infarction during influenza season and after angioplasty after 1 year of follow-up (HR = 0.59, 95% CI: 0.4–0.86, *P* = 0.004) (21). The FLUCAD study, which was published in 2008 and included 658 optimally treated CAD patients, found that the influenza vaccine improved the clinical prognosis of patients with coronary artery disease (HR = 0.54, 95% CI: 0.24–1.21, *P* = 0.13) and reduced the frequency of coronary ischemic events in nearly 1 year of follow-up (HR = 0.54, 95% CI: 0.29–0.99, *P* = 0.047) (22). Another randomized trial, which was published in 2011 and included 439 patients admitted to acute coronary syndromes (ACS) within 8 weeks, also found that the influenza vaccine reduced the number of major cardiovascular events in patients with ACS after a 1-year follow-up (unadjusted HR = 0.70, 95% CI: 0.57–0.86, *P* = 0.004) (23). A meta-analysis based on six randomized controlled studies published in 2022 also found that the influenza vaccine was associated with reduced cardiovascular risk (24), and two meta-analyses also published in 2022 further suggested that the influenza vaccine was associated with reduced cardiovascular risk in patients with heart failure (25) or coronary artery disease (26). This review shows an expanded effect of influenza vaccination on the risk of arrhythmias. Importantly, our results also suggest a statistically inverse association between the influenza vaccine and the risk of AF. As the most common cardiac arrhythmia in clinical practice, the incidence of AF largely increases with age, significantly contributing to ischemic stroke, heart failure, morbidity, and mortality (27). Therefore, our results might provide novel insight into AF prevention.

### Underlying mechanism

**FIGURE 4:**
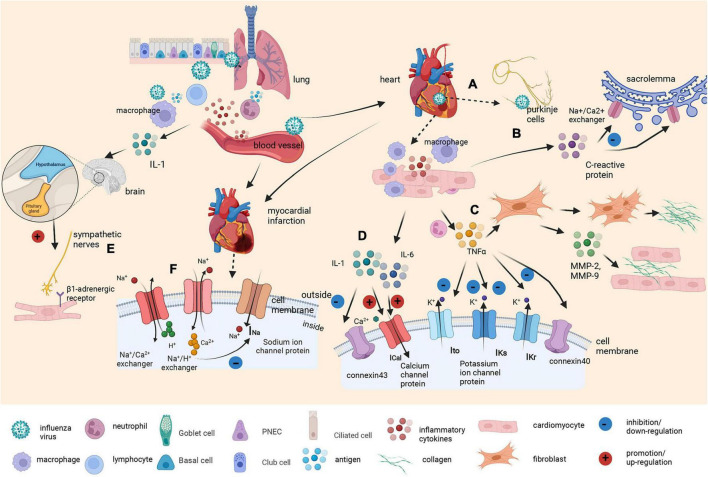
Graphical abstract on the underlying molecular mechanism of the influenza virus as a trigger of arrhythmia. Influenza viruses enter the body through the respiratory tract. The innate immune response induces various cytokines and chemokines to recruit macrophages and neutrophils to control the virus. Inflammatory cytokines also increase vascular permeability, which helps the virus enter the bloodstream. **(A)** Once the virus reaches the heart, it can replicate in cardiomyocytes and Purkinje cells, directly affecting the cardiac conduction system. **(B)** At the same time, elevated C-reactive protein levels leads to membrane dysfunction by inhibiting the exchange of sodium and calcium ions in sacrolemmal vesicles. **(C)** Tumor necrosis factor α (TNFα) produced by macrophages surrounding cardiomyocytes promotes fibroblast activation and collagen III deposition. It also induces proliferation, the secretion of matrix metalloproteinase-2 (MMP-2) and matrix metalloproteinase-9 (MMP-9), and the invasion of myocardial fibroblasts. Additionally, it reduces the expression of connexin40, results in a strong decrease in the transient outward current (Ito) and downregulates the rapid and slow component of the delayed rectifier potassium current (IKr and IKs). **(D)** Other cytokines, such as interleukin-6 (IL-6) and interleukin-1β (IL-1β), prolong APD by enhancing the L-type calcium current (ICaL). IL-1β also reduces connexin 43 (Cx43) expression to a certain extent. **(E)** Viral infection also acts as a stressor to activate the hypothalamic–pituitary–adrenal axis by stimulating macrophages to produce IL-1. Cardiomyocyte β1-adrenergic receptor activation intricately affects calcium and potassium conductance. **(F)** Infection with the influenza virus also affects the metabolism of cardiomyocytes. Inflammatory cytokines and CRP induced by influenza infection may cause the occurrence and development of myocardial ischemia and myocarditis. The accumulation of ADP and anaerobic glycolytic products, including lactic acid and ATP-derived hydrogen ions, leads to intracellular acidification, which activates the Na^+^/H^+^ exchanger, causing H^+^ transport out of the cell in exchange for Na^+^ transport into the cell (resulting in increased intracellular Na^+^) and resulting in the activation of Na^+^/Ca^2+^ exchangers operating in the opposite mode (Na^+^ is transported out in exchange for Ca^2+^). This, in turn, causes cell swelling and Ca^2+^ overload, leading to electrophysiological disturbances, including membrane depolarization that contributes to the inactivation of Na^+^ channels and a reduction in fast Na^+^ currents.

Although the exact mechanism remains to be elucidated, there are several possible explanations (Figure 4). The mechanisms are mainly divided into direct and indirect effects. Previous animal studies have shown that the influenza virus can replicate in cardiomyocytes and Purkinje cells, directly affecting the cardiac conduction system (28). On the other hand, flu virus can also indirectly affect the cardiac conduction system. First, it indirectly affects ion channels in cardiomyocytes. Influenza virus infection has been shown to upregulate interleukin-6 (IL-6), interleukin-1β (IL-1β), tumor necrosis factor α (TNFα), and matrix metalloproteinases (MMPs) in the myocardium (29, 30). Increased TNFα expression results in a strong decrease in the transient outward potassium current (Ito) and a corresponding decrease in potassium channel protein expression (31–33). In addition, TNFα downregulates the rapid component of the delayed rectifier potassium current (IKr) by impairing human ether-a-go-go-related gene (hERG) potassium channel function (34), while IL-6 and IL-1 also prolong action potential duration (APD) by enhancing the L-type calcium current (ICaL) (35, 36). The result is a prolonged APD of cardiomyocytes, which leads to VAs.

Second, the flu virus induces arrhythmias by remodeling the structure of the heart and the connections between cells. TNFα promotes fibroblast activation, leading to collagen III deposition and eventually contributing to ventricular and atrial fibrosis (37). TNFα also downregulates the expression of gap junction protein Cx40 or Cx43, which are major connexin proteins in the myocardium (38). Third, virus infection can act as a stressor and activate the hypothalamic–pituitary–adrenal axis by stimulating macrophages to produce IL-1 (39). Activation of the sympathetic nervous system affects not only the immune system but also all systems under its control, including the cardiac system (40). Cardiomyocyte β1-adrenergic receptor activation intricately affects calcium and potassium conductance, contributing to the onset of arrhythmias (41).

Last, infection with the influenza virus also affects the metabolism of cardiomyocytes. Studies have shown that inflammatory cytokines and CRP induced by influenza infection are also related to the occurrence and aggravation of myocardial ischemia, especially in individuals with comorbidities (42–45). The depletion of intracellular adenosine-triphosphate (ATP) coupled with the accumulation of adenosine-diphosphate (ADP) and anaerobic glycolytic products, including lactic acid and ATP-derived hydrogen ions, leads to intracellular acidification, which activates the Na^+^/H^+^ exchanger, causing H^+^ transport out of the cell in exchange for Na transport into the cell (resulting in increased intracellular Na). This, in turn, causes cell swelling and Ca^2+^ overload, which is secondary to the activation of Na^+^/Ca^2+^ exchangers operating in the opposite mode (Na^+^ is transported out in exchange for Ca^2+^). These metabolic changes are accompanied by electrophysiological disturbances, including membrane depolarization that causes the inactivation of Na^+^ channels and a reduction in fast Na^+^ currents, consequently leading to a slowing of conduction that can result in subsequent arrhythmias.

### Implications for further study

The results of this review suggest a positive effect of influenza vaccination on the risk of arrhythmia. Furthermore, the 2006 American and 2019 European secondary cardiovascular prevention guidelines recommend influenza vaccinations (class I, level of evidence B recommendation) to prevent influenza infection from exacerbating the condition of patients with cardiovascular disease (15, 46, 47). Our results reinforced the current guidelines. Therefore, clinicians should encourage patients with high-risk cardiovascular diseases, such as elderly adults and patients with smoking habits, obesity, diabetes, obstructive sleep apnea, and hypertension, to receive influenza vaccination annually to prevent arrhythmia (48). Further studies would assess the benefit of influenza vaccination against arrhythmias in individuals at low risk for cardiovascular diseases.

### Study limitations

Our study has several limitations. First, most of the included studies were observational, and therefore, we cannot prove causality. Additionally, the most articles that we included were retrospective cohort studies and case-control studies. The benefit of influenza vaccination on arrhythmia were not significant in the subgroup analysis, which may be limited by the reduced sample size. More prospective studies may be needed to confirm the effect of the flu vaccine on the risk of arrhythmias. Second, all the observational studies were retrospective, and recall bias is unavoidable. Furthermore, some studies did not adjust for several clinical confounders, such as age or comorbidities. Third, substantial heterogeneity was found in the main results. When stratified by arrhythmia type, the heterogeneity was reduced to 0, but it remained moderate in VAs. The difference in the baseline clinical characteristics across studies, such as age, comorbidities, the measurement of VA, and study design, might be responsible for the heterogeneity. Finally, the population included in this article had different health conditions, and the participants were highly heterogeneous. Further studies are needed to investigate the effects of the flu vaccine on the risk of arrhythmias in people with different health conditions, especially patients with CVD.

## Conclusion

Based on the current evidence, we found that influenza vaccination may be linked to a reduced risk of arrhythmias, especially AF. Our results need to be interpreted with caution, and more prospective studies are needed to confirm our findings. Moreover, the effect of influenza vaccination on the risk of VAs and arrhythmias in individuals at low risk for cardiovascular diseases should be further studied.

## Data availability statement

The original contributions presented in this study are included in the article/Supplementary material, further inquiries can be directed to the corresponding authors.

## Author contributions

ML and ZY contributed to the study concept and design and revised the draft. WL, PY, and YZ performed the search strategy and contributed to database research, acquisition of data, and statistical analyses. All the authors participated in data analysis, reviewed, and approved the final manuscript.

## Footnotes

1
http://www.prisma-statement.org

## Abbreviations

RCT: randomized controlled trial
OR: odds ratio
CI: confidence interval
CAEFISS: Canadian Adverse Events Following Immunization Surveillance System
PRISMA: preferred reporting items for systematic reviews and meta-analyses
RRs: relative risks
HRs: hazard ratios
PICOS: population, intervention, comparison, outcome, and study design
NOS: Newcastle-Ottawa Scale
RevMan: Review Manager
AF: atrial fibrillation
ICD: implantable cardioverter defibrillator
aHR: adjusted hazard ratio
VA: ventricular arrhythmia
FLUVACS study: Flu vaccination in acute coronary syndromes and planned percutaneous coronary interventions Study
FLUCAD study: Influenza vaccination in secondary prevention from coronary ischaemic events in coronary artery disease Study
ACS: acute coronary syndromes
IL-6: interleukin-6
IL-1 β: interleukin-1 β
TNF α: tumor necrosis factor α
MMPs: matrix metalloproteinases.
