# Supplementary material for: Influenza vaccination is associated with a decreased risk of atrial fibrillation: A systematic review and meta-analysis
Source: Front Cardiovasc Med. 2022 Oct 20;9:970533. doi: 10.3389/fcvm.2022.970533 (PMC9630361; doi:10.3389/fcvm.2022.970533)
Supplement: Supplementary file 1 [file Data_Sheet_1.docx]

# Supplementary Content

Influenza vaccination is associated with decreased risk of atrial fibrillation: A systematic review and meta-analysis

**Table S1. PRISMA Checklist.**

**Table S2: Studies excluded (n=17) with reasons.**

**Table S3: Quality assessment of the included observational studies by Newcastle–Ottawa scale.**

**Methods: Search Terms, Data Sources and Detailed Search Strategy.**

**Figure S1. Egger’s test and Begg’s test demonstrating potential publication bias of included studies that reported the association.**

**Figure S2. Forest plot for the association between influenza vaccination and the risk of arrhythmia, stratified by study design.**

This supplementary material has been provided by the authors to give readers additional information about their work.

**Table S1. PRISMA Checklist.**

| **Section and Topic** | **Item #** | **Checklist item** | **Location where item is reported** |
| --- | --- | --- | --- |
| **TITLE** | | |  |
| Title | 1 | Identify the report as a systematic review. | 1 |
| **ABSTRACT** | | |  |
| Abstract | 2 | See the PRISMA 2020 for Abstracts checklist. | 2 |
| **INTRODUCTION** | | |  |
| Rationale | 3 | Describe the rationale for the review in the context of existing knowledge. | 3 |
| Objectives | 4 | Provide an explicit statement of the objective(s) or question(s) the review addresses. | 3 |
| **METHODS** | | |  |
| Eligibility criteria | 5 | Specify the inclusion and exclusion criteria for the review and how studies were grouped for the syntheses. | 4-5 |
| Information sources | 6 | Specify all databases, registers, websites, organisations, reference lists and other sources searched or consulted to identify studies. Specify the date when each source was last searched or consulted. | 4 |
| Search strategy | 7 | Present the full search strategies for all databases, registers and websites, including any filters and limits used. | 4 |
| Selection process | 8 | Specify the methods used to decide whether a study met the inclusion criteria of the review, including how many reviewers screened each record and each report retrieved, whether they worked independently, and if applicable, details of automation tools used in the process. | 5 |
| Data collection process | 9 | Specify the methods used to collect data from reports, including how many reviewers collected data from each report, whether they worked independently, any processes for obtaining or confirming data from study investigators, and if applicable, details of automation tools used in the process. | 5 |
| Data items | 10a | List and define all outcomes for which data were sought. Specify whether all results that were compatible with each outcome domain in each study were sought (e.g. for all measures, time points, analyses), and if not, the methods used to decide which results to collect. | 5 |
|  | 10b | List and define all other variables for which data were sought (e.g. participant and intervention characteristics, funding sources). Describe any assumptions made about any missing or unclear information. | 5 |
| Study risk of bias assessment | 11 | Specify the methods used to assess risk of bias in the included studies, including details of the tool(s) used, how many reviewers assessed each study and whether they worked independently, and if applicable, details of automation tools used in the process. | 5 |
| Effect measures | 12 | Specify for each outcome the effect measure(s) (e.g. risk ratio, mean difference) used in the synthesis or presentation of results. | 5 |
| Synthesis methods | 13a | Describe the processes used to decide which studies were eligible for each synthesis (e.g. tabulating the study intervention characteristics and comparing against the planned groups for each synthesis (item #5)). | 5 |
|  | 13b | Describe any methods required to prepare the data for presentation or synthesis, such as handling of missing summary statistics, or data conversions. | 5 |
|  | 13c | Describe any methods used to tabulate or visually display results of individual studies and syntheses. | 5 |
|  | 13d | Describe any methods used to synthesize results and provide a rationale for the choice(s). If meta-analysis was performed, describe the model(s), method(s) to identify the presence and extent of statistical heterogeneity, and software package(s) used. | 5 |
|  | 13e | Describe any methods used to explore possible causes of heterogeneity among study results (e.g. subgroup analysis, meta-regression). | 5 |
|  | 13f | Describe any sensitivity analyses conducted to assess robustness of the synthesized results. | 5 |
| Reporting bias assessment | 14 | Describe any methods used to assess risk of bias due to missing results in a synthesis (arising from reporting biases). | 5 |
| Certainty assessment | 15 | Describe any methods used to assess certainty (or confidence) in the body of evidence for an outcome. | 5 |
| **RESULTS** | | |  |
| Study selection | 16a | Describe the results of the search and selection process, from the number of records identified in the search to the number of studies included in the review, ideally using a flow diagram. | 5-6 |
|  | 16b | Cite studies that might appear to meet the inclusion criteria, but which were excluded, and explain why they were excluded. | 6 |
| Study characteristics | 17 | Cite each included study and present its characteristics. | 6 |
| Risk of bias in studies | 18 | Present assessments of risk of bias for each included study. | 7 |
| Results of individual studies | 19 | For all outcomes, present, for each study: (a) summary statistics for each group (where appropriate) and (b) an effect estimate and its precision (e.g. confidence/credible interval), ideally using structured tables or plots. | 6-7 |
| Results of syntheses | 20a | For each synthesis, briefly summarise the characteristics and risk of bias among contributing studies. | 7 |
|  | 20b | Present results of all statistical syntheses conducted. If meta-analysis was done, present for each the summary estimate and its precision (e.g. confidence/credible interval) and measures of statistical heterogeneity. If comparing groups, describe the direction of the effect. | 7 |
|  | 20c | Present results of all investigations of possible causes of heterogeneity among study results. | 7 |
|  | 20d | Present results of all sensitivity analyses conducted to assess the robustness of the synthesized results. | 7 |
| Reporting biases | 21 | Present assessments of risk of bias due to missing results (arising from reporting biases) for each synthesis assessed. | 7 |
| Certainty of evidence | 22 | Present assessments of certainty (or confidence) in the body of evidence for each outcome assessed. | 7 |
| **DISCUSSION** | | |  |
| Discussion | 23a | Provide a general interpretation of the results in the context of other evidence. | 7-8 |
|  | 23b | Discuss any limitations of the evidence included in the review. | 12 |
|  | 23c | Discuss any limitations of the review processes used. | 12 |
|  | 23d | Discuss implications of the results for practice, policy, and future research. | 11 |
| **OTHER INFORMATION** | | |  |
| Registration and protocol | 24a | Provide registration information for the review, including register name and registration number, or state that the review was not registered. | 4 |
|  | 24b | Indicate where the review protocol can be accessed, or state that a protocol was not prepared. | 4 |
|  | 24c | Describe and explain any amendments to information provided at registration or in the protocol. | 4 |
| Support | 25 | Describe sources of financial or non-financial support for the review, and the role of the funders or sponsors in the review. | 13 |
| Competing interests | 26 | Declare any competing interests of review authors. | 13 |
| Availability of data, code and other materials | 27 | Report which of the following are publicly available and where they can be found: template data collection forms; data extracted from included studies; data used for all analyses; analytic code; any other materials used in the review. | 13 |

*From:*  Page MJ, McKenzie JE, Bossuyt PM, Boutron I, Hoffmann TC, Mulrow CD, et al. The PRISMA 2020 statement: an updated guideline for reporting systematic reviews. BMJ 2021;372:n71. doi: 10.1136/bmj.n71

**Table S2: Studies excluded (n=17) with reasons**

| **Studies excluded** | **Reasons** |
| --- | --- |
| Clar,2015[1] | This is a review |
| Kaya,2017[2] | Not the target outcome: CV death、HFrH and recurrent HFrH |
| Meyers,2003[3] | This is a review |
| Awadalla,2019[4] | The clinical heterogeneity is high. |
| Christiansen,2019[5] | Not the target outcome: 1-year risk of myocardial infarction, stroke, heart failure, pneumonia, and mortality |
| Fountoulaki,2018[6] | This is a review |
| Grau,2006[7] | This is a review |
| Kao,2017[8] | Not the target outcome: ischemic stroke |
| Lapi,2019[9] | Not the target exposure: adjuvanted versus nonadjuvanted influenza vaccines |
| Liu,2017[10] | Not the target outcome: hemorrhagic stroke |
| McCarthy,2016[11] | Not the target outcome: 30-day mortality |
| Tsai,,2020[12] | Not the targeted outcomes: hospitalization, pneumonia, circulatory conditions, critical illness, and death |
| Ma,2020[13] | Not the target exposure: received both PPV and influenza vaccine vesus received no vaccine |
| Devore,2019[14] | This is a review |
| Meyers,2003[15] | This is a review |
| Pérez-Rubio,2021[16] | This is a review |
| Rodrigues,2021[17] | This is a review |

1. Clar, C., et al., *Influenza vaccines for preventing cardiovascular disease.* Cochrane Database of Systematic Reviews, 2015(5).

2. Kaya, H., et al., *Influence of influenza vaccination on recurrent hospitalization in patients with heart failure.* Herz, 2017. **42**(3): p. 307-315.

3. Meyers, D.G., *Myocardial infarction, stroke, and sudden cardiac death may be prevented by influenza vaccination.* Curr Atheroscler Rep, 2003. **5**(2): p. 146-9.

4. Awadalla, M., et al., *Influenza vaccination and myocarditis among patients receiving immune checkpoint inhibitors.* J Immunother Cancer, 2019. **7**(1): p. 53.

5. Christiansen, C.F., et al., *Influenza vaccination and 1-year risk of myocardial infarction, stroke, heart failure, pneumonia, and mortality among intensive care unit survivors aged 65 years or older: a nationwide population-based cohort study.* Intensive Care Medicine, 2019. **45**(7): p. 957-967.

6. Fountoulaki, K., et al., *Beneficial effects of vaccination on cardiovascular events: Myocardial infarction, stroke, heart failure.* Cardiology (Switzerland), 2018. **141**(2): p. 98-106.

7. Grau, A.J., L. Marquardt, and C. Lichy, *The effect of infections and vaccinations on stroke risk.* Expert Review of Neurotherapeutics, 2006. **6**(2): p. 175-183.

8. Kao, P.F., et al., *Influenza vaccination might reduce the risk of ischemic stroke in patients with atrial fibrillation: A population-based cohort study.* Oncotarget, 2017. **8**(68): p. 112697-112711.

9. Lapi, F., et al., *Adjuvanted versus nonadjuvanted influenza vaccines and risk of hospitalizations for pneumonia and cerebro/cardiovascular events in the elderly.* Expert Review of Vaccines, 2019. **18**(6): p. 663-670.

10. Liu, J.C., et al., *Influenza vaccination reduces hemorrhagic stroke risk in patients with atrial fibrillation: A population-based cohort study.* Int J Cardiol, 2017. **232**: p. 315-323.

11. McCarthy, N.L., et al., *Vaccination and 30-day mortality risk in children, adolescents, and young adults.* Pediatrics, 2016. **137**(3).

12. Tsai, L.W., et al., *Statin use and influenza vaccine effectiveness in persons >65 years of age, Taiwan.* Emerging Infectious Diseases, 2020. **26**(6): p. 1243-1250.

13. Ma, J., et al., *TCT CONNECT-220 Influenza and Pneumonia Vaccination Effect on Cardiovascular Events.* Journal of the American College of Cardiology, 2020. **76**(17): p. B95-B96.

14. Devore, A.D. and A.S. Bhatt, *Influenza Vaccination in Patients With Heart Failure: Time to Give It Our Best Shot.* Circulation, 2019. **139**(5): p. 587-589.

15. Meyers, D.G., *Could influenza vaccination prevent myocardial infarction, stroke and sudden cardiac death?* Am J Cardiovasc Drugs, 2003. **3**(4): p. 241-4.

16. Pérez-Rubio, A., J.A. San Román, and J.M. Eiros Bouza, *The impact of influenza vaccination on cardiovascular disease.* Med Clin (Barc), 2021. **157**(1): p. 22-32.

17. Rodrigues, B.S., et al., *The impact of influenza vaccination in patients with cardiovascular disease: An overview of systematic reviews.* Trends in Cardiovascular Medicine, 2021. **31**(5): p. 315-320.

**Table S3 Quality assessment of the included observational studies by Newcastle–Ottawa scale.**

1. **Cohort study**

| Author  (Publication Year) | Newcastle-Ottawa Scale | | | | | | | | | |
| --- | --- | --- | --- | --- | --- | --- | --- | --- | --- | --- |
|  | Selection | | | Comparability | | | Outcome | | | Total |
|  | a | b | c | d | e | f | g | h | i |  |
| Chen, 2021 | 1 | 1 | 1 | 0 | 1 | 1 | 0 | 1 | 0 | 6 |
| McNeil, 2019 | 1 | 1 | 1 | 1 | 1 | 1 | 1 | 1 | 1 | 9 |
| Modin, 2018 | 1 | 1 | 1 | 0 | 1 | 1 | 1 | 1 | 1 | 8 |
| singh,2015 | 1 | 1 | 1 | 0 | 1 | 1 | 1 | 0 | 1 | 7 |

1. Representativeness of the exposed cohort.
2. Selection of the non-exposed cohort.
3. Ascertainment of exposure.
4. Demonstration that outcome of interest was not present at start of study.
5. Comparability of cohorts on the basis of the design or analysis (adjusted for age).
6. Comparability of cohorts on the basis of the design or analysis (adjusted for any other factor).
7. Assessment of outcome.
8. Was follow-up long enough for outcomes to occur. (At least 1 year to eliminate seasonal effects of influenza).
9. Adequacy of follow-up of cohorts.
10. **Case-control study**

| Author  (Publication Year) | Newcastle-Ottawa Scale | | | | | | | | | |
| --- | --- | --- | --- | --- | --- | --- | --- | --- | --- | --- |
|  | Selection | | | Comparability | | | Exposure | | | Total |
|  | a | b | c | d | e | f | g | h | i |  |
| Chang, 2016 | 1 | 1 | 1 | 1 | 1 | 1 | 1 | 1 | 1 | 9 |
| Siscovick, 2000 | 1 | 1 | 1 | 1 | 1 | 1 | 1 | 1 | 0 | 8 |

1. Definition of cases.
2. Representativeness of the cases.
3. Selection of Controls.
4. Definition of Controls.
5. Comparability of cases and controls on the basis of the design or analysis (study controls for age).
6. Comparability of cases and controls on the basis of the design or analysis (adjusted for any other factor).
7. Ascertainment of exposure.
8. Same method of ascertainment for cases and controls.
9. Non-Response rate.

**Methods: Search Terms, Data Sources and Detailed Search Strategy**

**Search terms**

The search terms were: “Arrhythmias”, “Atrial Fibrillation”, “Atrial Flutter”, “Ventricular Fibrillation”, “Ventricular Flutter”, “Ventricular Tachycardia”, “Heart Arrest”, “Influenza vaccination)”.

**Data sources:**

The PubMed, Embase, and Cochrane Library databases were searched to identify studies that investigated the potential effects of the influenza vaccine on arrhythmia risk that were published until October 25th, 2021. We did not restrict the search by language or date of publication. And we also check the conference abstracts and bibliographies of related literature to obtain other articles that might meet the requirements.

**Detailed search strategy**

Our final combined **Pubmed** Search (https://pubmed.ncbi.nlm.nih.gov/advanced/) strategy was as follows:

(((((((arrhythmias, cardiac[MeSH Terms]) OR (Arrhythmia, Cardiac)) OR (Cardiac Dysrhythmia)) OR (Dysrhythmia, Cardiac)) OR (Cardiac Arrhythmia)) OR (Cardiac Arrhythmias)) OR (Arrhythmia)) OR (Arrythmia).

Search Strategy for Arrhythmias.

OR

(((((((((((((((((((((((((Atrial Fibrillation[MeSH Terms]) OR (Atrial Fibrillations)) OR (Fibrillation, Atrial)) OR (Auricular Fibrillation)) OR (Auricular Fibrillations)) OR (Fibrillation, Auricular)) OR (Fibrillations, Auricular)) OR (Persistent Atrial Fibrillation)) OR (Atrial Fibrillation, Persistent)) OR (Atrial Fibrillations, Persistent)) OR (Fibrillation, Persistent Atrial)) OR (Fibrillations, Persistent Atrial)) OR (Persistent Atrial Fibrillations)) OR (Familial Atrial Fibrillation)) OR (Atrial Fibrillation, Familial)) OR (Atrial Fibrillations, Familial)) OR (Familial Atrial Fibrillations)) OR (Fibrillation, Familial Atrial)) OR (Fibrillations, Familial Atrial)) OR (Paroxysmal Atrial Fibrillation)) OR (Atrial Fibrillation, Paroxysmal)) OR (Atrial Fibrillations, Paroxysmal)) OR (Fibrillation, Paroxysmal Atrial)) OR (Fibrillations, Paroxysmal Atrial)) OR (Paroxysmal Atrial Fibrillations)) OR (Paroxysmal Atrial Fibrillations)

Search Strategy for Atrial Fibrillation.

OR

(((((((atrial flutter[MeSH Terms]) OR (Atrial Flutters)) OR (Flutter, Atrial)) OR (Flutters, Atrial)) OR (Auricular Flutter)) OR (Auricular Flutters)) OR (Flutter, Auricular)) OR (Flutters, Auricular)

Search Strategy for atrial flutter.

OR

(Ventricular flutter[MeSH Terms]) OR (Ventricular Flutters)

Search Strategy for Ventricular flutter

OR

(((Ventricular Fibrillation[MeSH Terms]) OR (Fibrillation, Ventricular)) OR (Fibrillations, Ventricular)) OR (Ventricular Fibrillations)

Search Strategy for Ventricular Fibrillation.

OR

((((((((((((((((((Tachycardia, Ventricular[MeSH Terms]) OR (Ventricular Tachycardias)) OR (Ventricular Tachyarrhythmias)) OR (Tachyarrhythmia, Ventricular)) OR (Ventricular Tachyarrhythmia)) OR (Ventricular Tachycardia)) OR (Nonsustained Ventricular Tachycardia)) OR (Nonsustained Ventricular Tachycardias)) OR (Tachycardia, Nonsustained Ventricular)) OR (Ventricular Tachycardia, Nonsustained)) OR (Paroxysmal Supraventricular Tachycardia)) OR (Paroxysmal Supraventricular Tachycardias)) OR (Supraventricular Tachycardia, Paroxysmal)) OR (Tachycardia, Paroxysmal Supraventricular)) OR (Idiopathic Ventricular Tachycardia)) OR (Idiopathic Ventricular Tachycardias)) OR (Tachycardia, Idiopathic Ventricular)) OR (Ventricular Tachycardia, Idiopathic)) OR (ventricular arrhythmias)

Search Strategy for Tachycardia, Ventricular.

OR

(((((((Heart Arrest[MeSH Terms]) OR (Arrest, Heart)) OR (Cardiac Arrest)) OR (Arrest, Cardiac)) OR (Asystole)) OR (Asystoles)) OR (Cardiopulmonary Arrest)) OR (Arrest, Cardiopulmonary)

Search Strategy for Heart Arrest.

AND

(Influenza vaccination)

Search Strategy for Influenza vaccination.

Our final **EMBASE** search (https://www.embase.com/#advancedSearch/default) strategy was as follows:

#1 ‘heart arrhythmia’/exp OR ‘arrhythmia’ OR ‘arrhythmias, cardiac’ OR ‘arrhytmia, heart’ OR ‘cardiac arrhythmia’ OR ‘cardiac arrhythmias’ OR ‘cardiac arrythmia’ OR ‘cardiac disrhythmia’ OR ‘cardiac dysrhythmia’ OR ‘ cardial arrhythmia’ OR ‘ectopic heart rhythm’ OR ‘ectopic rhythm’ OR ‘heart aberrant conduction’ OR ‘heart arrhytmia’ OR ‘heart arrythmia’ OR ‘heart dysrhythmia’ OR ‘heart ectopic beat’ OR ‘heart ectopic ventricle contraction’ OR ‘heart rhythm disorder’ OR ‘myocardial arrhythmia’

#2 ‘atrial fibrillation’/exp OR ‘atrium fibrillation’ OR ‘auricular fibrilation’ OR ‘auricular fibrillation’ OR ‘cardiac atrial fibrillation’ OR ‘cardiac atrium fibrillation’ OR ‘fibrillation, heart atrium’ OR ‘heart atrial fibrillation’ OR ‘heart atrium fibrillation’ OR ‘ heart fibrillation atrium’ OR ‘non-valvular atrial fibrillation’ OR ‘nonvalvular atrial fibrillation’

#3 ‘heart atrium flutter’/exp OR ‘atrial flutter’ OR ‘atrium flutter’ OR ‘atrium flutter, heart’ OR ‘auricular flutter’ OR ‘cardiac atrial flutter’ OR ‘cardiac atrium flutter’ OR ‘flutter, heart atrium’ OR ‘heart atrial flutter’ OR ‘ supraventricular flutter’

#4 ‘heart ventricle flutter’/exp OR ‘flutter, heart ventricle’ OR ‘ventricular flutter’

#5 ‘heart ventricle fibrillation’/exp OR ‘cardiac ventricle fibrillation’ OR ‘cardiac ventricular fibrillation’ OR ‘fibrillation, heart ventricle’ OR ‘heart ventricular fibrillation’ OR ‘ventricle fibrillation’ OR ‘ventricle fibrillation, heart’ OR ‘ventricular fibrillation’

#6 ‘heart ventricle tachycardia’/exp OR ‘accelerated idioventricular rhythm’ OR ‘cardiac ventricle tachycardia’ OR ‘cardiac ventricle tachycardy’ OR ‘cardiac ventricular tachycardia’ OR ‘cardiac ventricular tachycardy’ OR ‘heart ventricular tachycardia’ OR ‘heart ventricular tachycardy’ OR ‘idioventricular tachycardia’ OR ‘tachycardia, heart ventricle’ OR ‘tachycardia, ventricular’ OR ‘ventricle tachycardia’ OR ‘ventricular tachyarrhythmia’ OR ‘ventricular tachycardia’ OR ‘ventricular tachycardias’

#7 ‘heart arrest’/exp OR ‘arrest, heart’ OR ‘asystole’ OR ‘asystolia’ OR ‘asystoly’ OR ‘cardiac arrest’ OR ‘circulation arrest’ OR ‘circulatory arrest’ OR ‘heart arrest, induced’ OR ‘heart asystole’ OR ‘heart standstill’ OR ‘induced heart arrest’

#8 ‘Influenza vaccination’/exp OR ‘influenza immunisation’ OR ‘influenza immunization’ OR ‘influenza inoculation’ OR ‘vaccination, influenza’

#9 #1 and #2 and #3 and #4 and #5 and #6 and #7 and #8

Our final **Cochrane** search strategy (https://www.cochranelibrary.com/advanced-search) was as follows(including Cochrane Reviews, Cochrane Protocols, Trials):

#1 MeSH descriptor: [Arrhythmias, Cardiac] explode all trees

#2 (Arrhythmia, Cardiac) OR (Cardiac Dysrhythmia) OR (Dysrhythmia, Cardiac) OR (Cardiac Arrhythmia) OR (Cardiac Arrhythmias) OR (Arrhythmia) OR (Arrythmia)

#3 MeSH descriptor: [Atrial Fibrillation] explode all trees

#4 (Atrial Fibrillations) OR (Fibrillation, Atrial) OR (Fibrillations, Atrial) OR (Auricular Fibrillation) OR (Auricular Fibrillations) OR (Fibrillation, Auricular) OR (Fibrillations, Auricular) OR (Persistent Atrial Fibrillation) OR (Atrial Fibrillation, Persistent) OR (Atrial Fibrillations, Persistent) OR (Fibrillation, Persistent Atrial) OR (Fibrillations, Persistent Atrial) OR (Persistent Atrial Fibrillations) OR (Familial Atrial Fibrillation) OR (Atrial Fibrillation, Familial) OR (Atrial Fibrillations, Familial) OR (Familial Atrial Fibrillations) OR (Fibrillation, Familial Atrial) OR (Fibrillations, Familial Atrial) OR (Paroxysmal Atrial Fibrillation) OR (Atrial Fibrillation, Paroxysmal) OR (Atrial Fibrillations, Paroxysmal) OR (Fibrillation, Paroxysmal Atrial) OR (Fibrillations, Paroxysmal Atrial) OR (Paroxysmal Atrial Fibrillations)

#5 MeSH descriptor: [Atrial Flutter] explode all trees

#6 (Atrial Flutters) OR (Flutter, Atrial) OR (Flutters, Atrial) OR (Auricular Flutter) OR (Auricular Flutters) OR (Flutter, Auricular) OR (Flutters, Auricular)

#7 MeSH descriptor: [Ventricular Flutter] explode all trees

#8 Ventricular Flutters

#9 MeSH descriptor: [Ventricular Fibrillation] explode all trees

#10 (Fibrillation, Ventricular) OR (Fibrillations, Ventricular) OR (Ventricular Fibrillations)

#11 MeSH descriptor: [Tachycardia, Ventricular] explode all trees

#12 (Ventricular Tachycardias) OR (Ventricular Tachyarrhythmias) OR (Tachyarrhythmia, Ventricular) OR (Ventricular Tachyarrhythmia) OR (Ventricular Tachycardia) OR (Nonsustained Ventricular Tachycardia) OR (Nonsustained Ventricular Tachycardias) OR (Tachycardia, Nonsustained Ventricular) OR (Ventricular Tachycardia, Nonsustained) OR (Paroxysmal Supraventricular Tachycardia) OR (Paroxysmal Supraventricular Tachycardias) OR (Supraventricular Tachycardia, Paroxysmal) OR (Tachycardia, Paroxysmal Supraventricular) OR (Idiopathic Ventricular Tachycardia) OR (Idiopathic Ventricular Tachycardias) OR (Tachycardia, Idiopathic Ventricular) OR (Ventricular Tachycardia, Idiopathic) OR (ventricular arrhythmias)

#13 MeSH descriptor: [Heart Arrest] explode all trees

#14 (Arrest, Heart) OR (Cardiac Arrest) OR (Arrest, Cardiac) OR (Asystole) OR (Asystoles) OR (Cardiopulmonary Arrest) OR (Arrest, Cardiopulmonary)

#15 (Influenza vaccination)

#16 (#1 OR #2) AND (#3 OR #4) AND (#5 OR #6) AND (#7 OR #8) AND (#9 OR #10) AND (#11 OR #12) AND (#13 OR #14) AND #15

**Figure S1. Egger’s test(P=0.222) and Begg’s test (P=0.452) demonstrating potential publication bias of included studies that reported the association between** **influenza vaccination and risk of arrhythmia.**

**
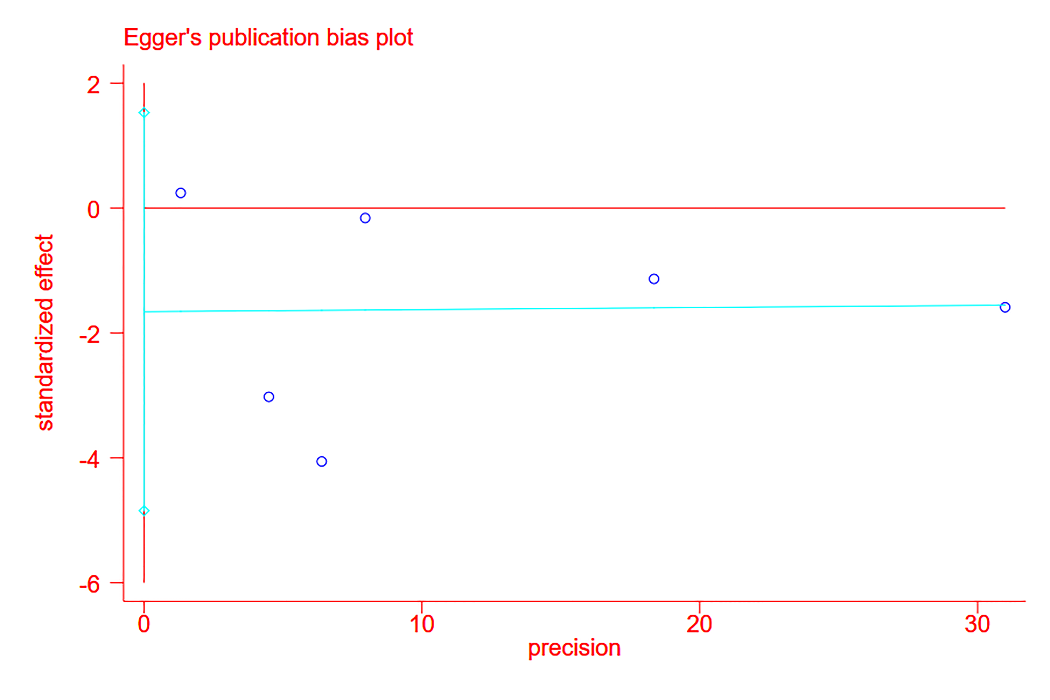

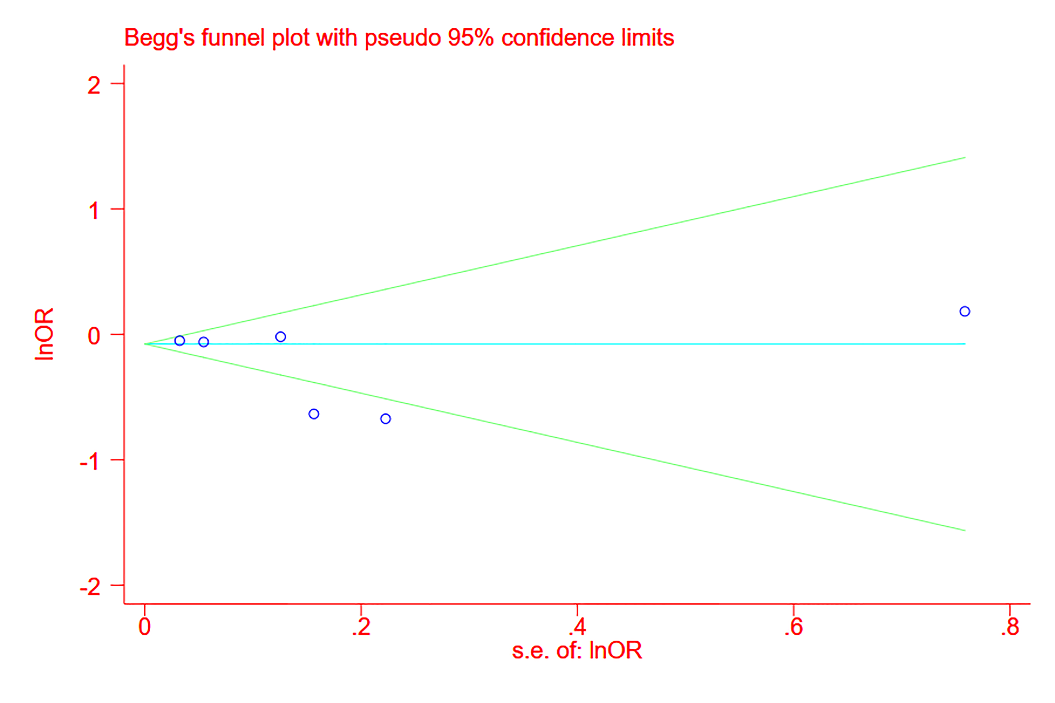
**

**Figure S2. Forest plot for the association between influenza vaccination and the risk of arrhythmia, stratified by study design.**

**
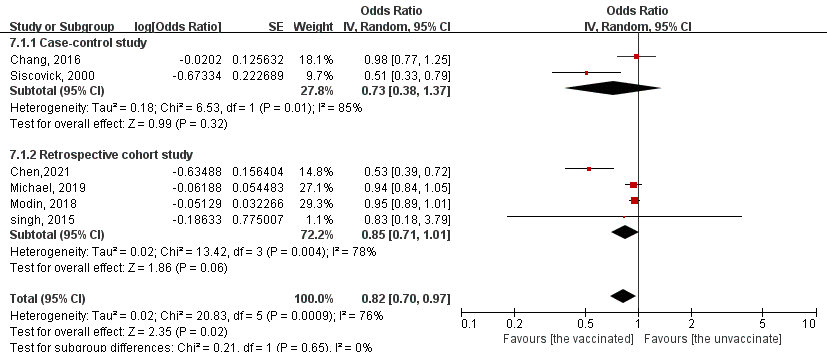
**
